# Non-synonymous FGD3 Variant as Positional Candidate for Disproportional Tall Stature Accounting for a Carcass Weight QTL (*CW-3*) and Skeletal Dysplasia in Japanese Black Cattle

**DOI:** 10.1371/journal.pgen.1005433

**Published:** 2015-08-25

**Authors:** Akiko Takasuga, Kunio Sato, Ryouichi Nakamura, Yosuke Saito, Shinji Sasaki, Takehito Tsuji, Akio Suzuki, Hiroshi Kobayashi, Tamako Matsuhashi, Koji Setoguchi, Hiroshi Okabe, Toshitake Ootsubo, Ichiro Tabuchi, Tatsuo Fujita, Naoto Watanabe, Takashi Hirano, Shota Nishimura, Toshio Watanabe, Makio Hayakawa, Yoshikazu Sugimoto, Takatoshi Kojima

**Affiliations:** 1 National Livestock Breeding Center, Odakura, Nishigo, Fukushima, Japan; 2 Shirakawa Institute of Animal Genetics, Japan Livestock Technology Association, Odakura, Nishigo, Fukushima, Japan; 3 Oita Prefectural Agriculture, Forestry and Fisheries Research Center, Kuju, Takeda, Oita, Japan; 4 Shimane Prefectural Livestock Technology Center, Koshi, Izumo, Shimane, Japan; 5 Miyagi Prefectural Livestock Experiment Station, Iwadeyama, Osaki, Miyagi, Japan; 6 Graduate School of Environmental and Life Science, Okayama University, Tsushima-naka, Okayama, Japan; 7 Aomori Prefectural Industrial Technology Research Center, Moritatukimino, Morita, Tugaru, Aomori, Japan; 8 Okayama Prefectural Research Institute of Livestock Industry, Misaki, Kume, Okayama, Japan; 9 Gifu Prefectural Livestock Research Institute, Kiyomi, Takayama, Gifu, Japan; 10 Cattle Breeding Development Institute of Kagoshima Prefecture, Osumi, So, Kagoshima, Japan; 11 Nagasaki Prefectural Beef Cattle Improvement Center, Tabiracho Kotedamen, Hirado, Nagasaki, Japan; 12 Saga Prefectural Livestock Experiment Station, Yamauchi, Takeo, Saga, Japan; 13 Tottori Animal Husbandry Experiment Station, Kotoura, Touhaku, Tottori, Japan; 14 School of Pharmacy, Tokyo University of Pharmacy and Life Science, Hachioji, Tokyo, Japan; University of Bern, SWITZERLAND

## Abstract

Recessive skeletal dysplasia, characterized by joint- and/or hip bone-enlargement, was mapped within the critical region for a major quantitative trait locus (QTL) influencing carcass weight; previously named *CW-3* in Japanese Black cattle. The risk allele was on the same chromosome as the *Q* allele that increases carcass weight. Phenotypic characterization revealed that the risk allele causes disproportional tall stature and bone size that increases carcass weight in heterozygous individuals but causes disproportionately narrow chest width in homozygotes. A non-synonymous variant of *FGD3* was identified as a positional candidate quantitative trait nucleotide (QTN) and the corresponding mutant protein showed reduced activity as a guanine nucleotide exchange factor for Cdc42. *FGD3* is expressed in the growth plate cartilage of femurs from bovine and mouse. Thus, loss of FDG3 activity may lead to subsequent loss of Cdc42 function. This would be consistent with the columnar disorganization of proliferating chondrocytes in chondrocyte-specific inactivated Cdc42 mutant mice. This is the first report showing association of *FGD3* with skeletal dysplasia.

## Author Summary

Livestock are typically subjected to intensive artificial selection for traits of economic value to producers. In spite of this strong selection, some major quantitative trait loci (QTLs) for an economically important trait never reach fixation in the population. Several studies have revealed that such QTLs are accompanied with an unfavorable effect on other traits of economic importance, including heritable disease phenotypes. The carcass weight QTL, named *CW-3*, was previously identified as one of three major QTL in Japanese Black cattle, and it was found to originate from a specific line that had been maintained in a regional subpopulation. Recent efforts to maintain genetic diversity of the Japanese Black breed have resulted in the widespread use of this line throughout Japan. Half-sib QTL analyses of the elite sires repeatedly detected the *CW-3* QTL, while skeletal dysplasia has been found in the descendants. Genomic analyses revealed that skeletal dysplasia is inseparably linked with *CW-3* and a functional variant of *FGD3* was identified as a positional candidate QTN. Further studies such as creating a genetically modified mouse model will be useful to understand a molecular mechanism of FGD3 to modulate bone development.

## Introduction

Carcass weight, as a measure of meat yield, is an economically important trait in livestock. Due to its economic significance, several quantitative trait locus (QTL) mapping and genome-wide association studies (GWAS) have been conducted with the objective of identifying genes for improving meat production. These studies reveal that body length or stature is often times directly related to meat yield [1–3]. In humans, adult height is a highly polygenic trait affected by several hundreds of loci [4], while some major loci have significant impact on body size in livestock [5,6]. In Japanese Black cattle, a previous GWAS detected three major loci for carcass weight [7]. Two loci, named *CW-1* and *CW-2*, correspond to *PLAG1* [7] and *NCAPG-LCORL* [2], respectively, both of which have been identified as loci influencing adult human height [8–10] and associated with body size-related traits in different cattle breeds and other livestock species (reviewed in [11]). The third locus, named *CW-3*, showed the largest allele substitution effect (+35.0 kg) among the three loci, while the *Q* allele frequency was the lowest (11.5%) and detected in a specific line of Japanese Black cattle [7].

A QTL allele with a large effect size that is not near fixation, may be associated with an unfavorable trait. Examples include the Lys-232-Ala substitution in *DGAT1* increases milk yield but reduces milk fat content in dairy cattle [12], and a frame-shift mutation in *MRC2* increases muscle mass in carriers but causes the recessive Crooked Tail Syndrome in Belgian Blue cattle [13]. In the case of *CW-3*, skeletal dysplasia is observed more frequently following line breeding within the founding genetic line. The disease is characterized by joint- and/or hip bone-enlargement but the conditions are various.

Here we show that *CW-3* is linked inseparably with skeletal dysplasia and a non-synonymous variant of *FGD3* causing a reduced activity of the encoding protein is a positional candidate QTN. The risk allele for skeletal dysplasia increased carcass weight in heterozygotes, most likely because of their taller stature and increased bone mass, while the homozygotes showed disproportionately narrow chest width causing an economic loss. The results explain the low *CW-3 Q* allele frequency despite its large effect on carcass weight.

## Results

### QTL mapping, identical-by-descent and haplotype-based association analyses identified a 3.3-Mb interval for a carcass weight QTL (*CW-3*) on chromosome 8

**Fig 1:**
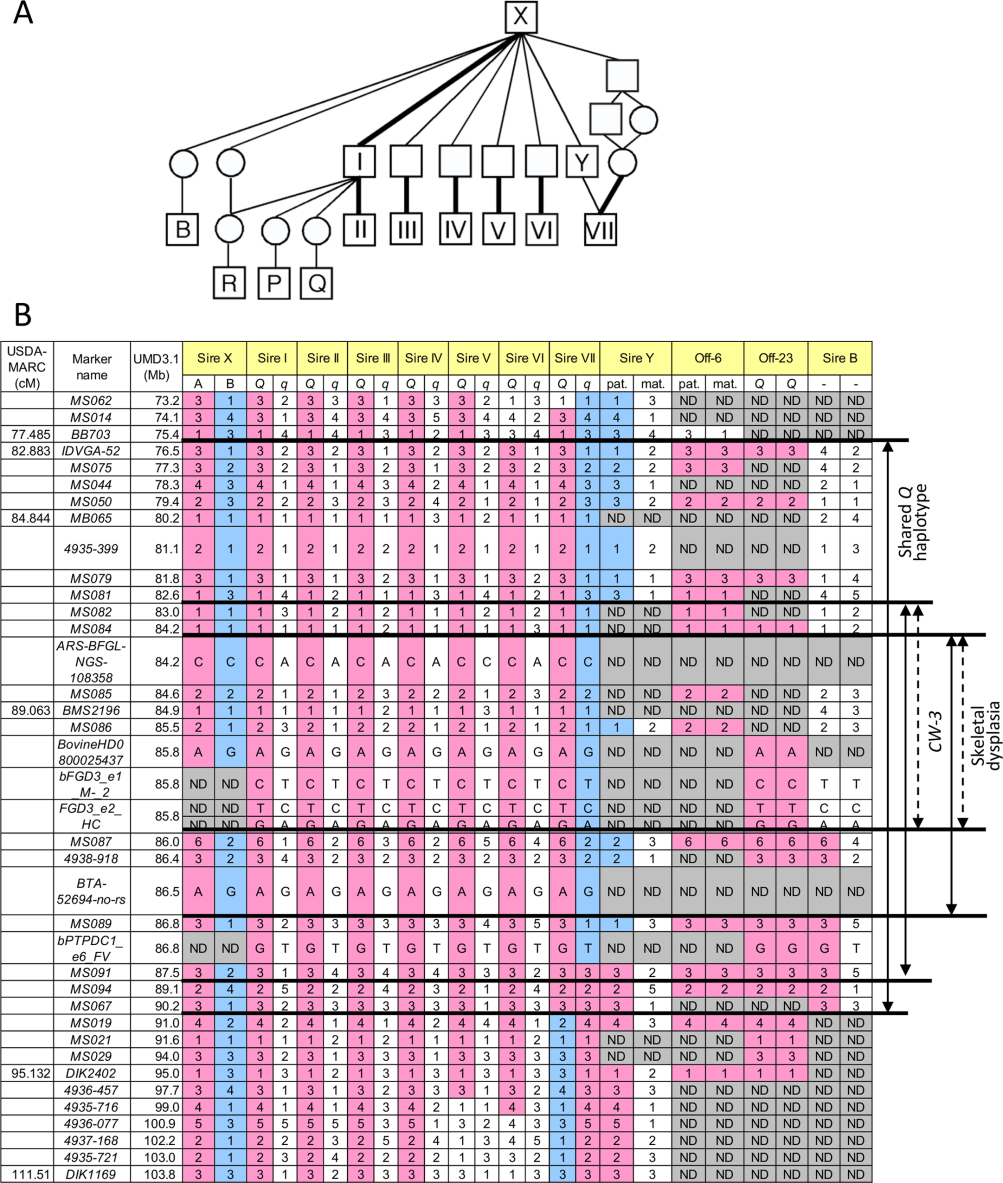
Pedigree and identical-by-descent (IBD) analysis. (A) A pedigree of *CW-3*-segregating (I-VII) and related sires. Bold lines indicate inheritance of the *Q* haplotype. (B) Haplotypes of *CW-3*-segregating sires and related animals. Sire B did not segregate *CW-3* nor produced affected offspring, strongly suggesting that the telomeric ends of the critical regions are at *MS087*. Marker information is shown in S7 Table. ND, not determined.

The *CW-3* QTL on bovine chromosome (BTA) 8 has been detected in seven Japanese Black paternal half-sib families of Sires I through VII (Fig 1A), of which two (Sires I and II) were described previously [7]. An allele substitution effect ranged from +21.1 kg to +40.1 kg for carcass weight and the QTL explained 4.5% to 18.6% of the phenotypic variance within respective families (S1 Table). The minimum overlapping region of the 95% confidence intervals (CI) for the QTL position was between *MB065* (80.2 Mb, UMD3.1) and *DIK2402* (95.0 Mb) (S1 Fig). Furthermore, the seven sires had a common ancestor, Sire X (Fig 1A), and shared a (hypothetical) identical-by-descent (IBD) *Q* haplotype between *IDVGA-52* (76.5 Mb) and *MS067* (90.2 Mb) (Fig 1B). The inheritance of the *Q* chromosome from Sire X to Sires I and II was evident (Fig 1). Interestingly, the *q* chromosome of Sire VII was also inherited from Sire X through Sire Y, and had a recombination event between *MS089* (86.8 Mb) and *MS091* (87.5 Mb); thus, the telomeric region distal to *MS091* was identical to the *Q* chromosomes of Sires I and II (Fig 1). These results identify the telomeric end of the QTL interval at *MS091* (87.5 Mb). On the other hand, the centromeric end of the QTL region was tentatively and conservatively determined at *IDVGA-52* (76.5 Mb) to include not only one (Sire V, between *MB065* and *DIK1169* for body weight) (S1 Fig) but also another 95% CI (Sire II, between *IDVGA-52* and *DIK2402*) [7]. The resultant critical region was an 11-Mb interval from *IDVGA-52* (76.5 Mb) to *MS091* (87.5 Mb), which was covered by a shared *Q* haplotype (Fig 1B).

**Fig 2:**
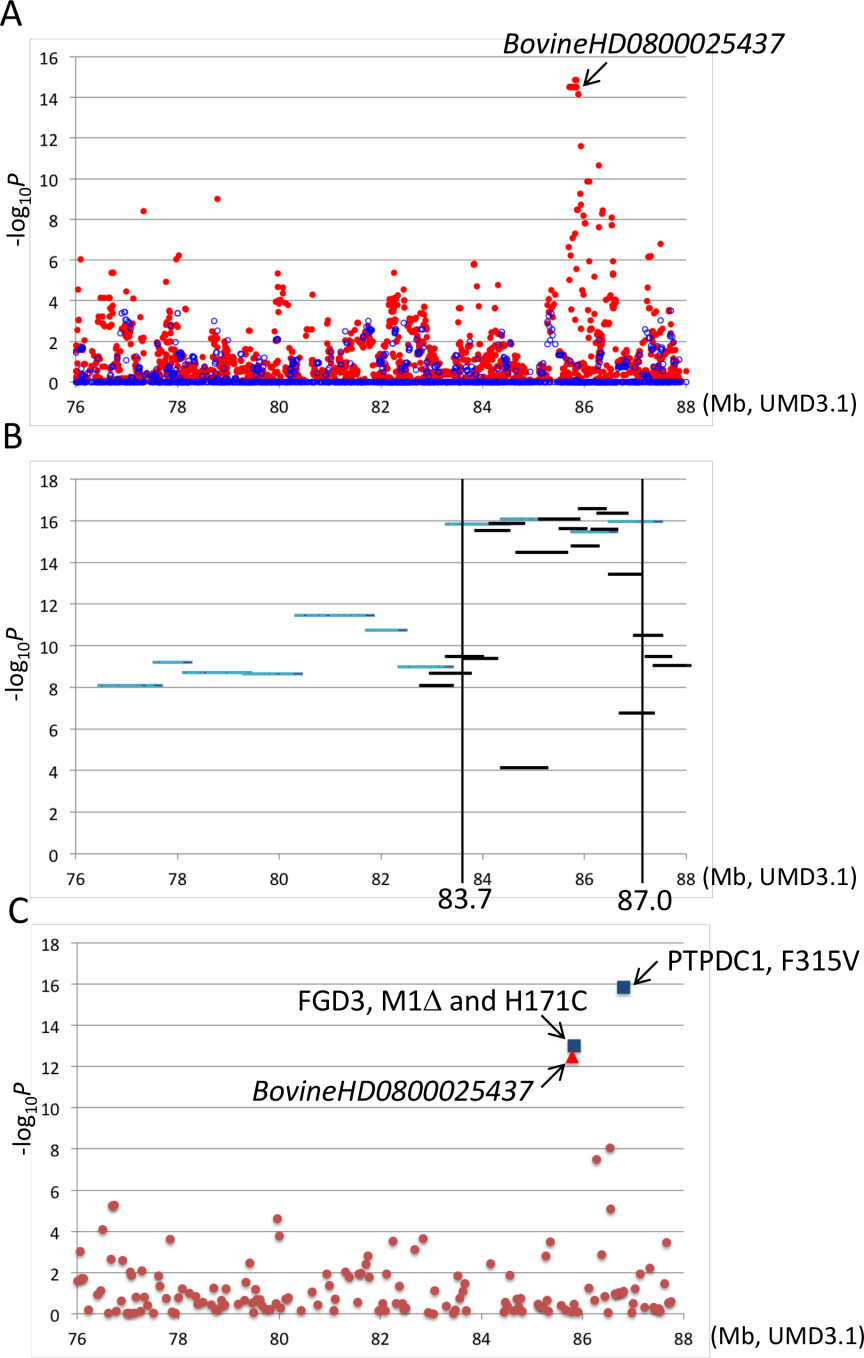
Association analyses using imputed BovineHD genotypes (A), segments of the identical-by-descent (IBD) *Q* haplotype (B), and candidate causative variations (C). Association with carcass weight was analyzed by a variance component approach using EMMAX software [39] with adjustments for age, slaughterhouse, and year as covariates and fixed effects. (A) Red and blue dots represent *p* values of imputed BovineHD genotypes in–log_10_ scale before and after conditioning, respectively. A conditioned analysis was performed by including an imputed genotype of *BovineHD0800025437* as a covariate in the model. (B) Light blue and black lines represent *p* values of approximately 1-Mb and 500-kb *Q* haplotypes in–log_10_ scale, respectively. (C) Brown dots, a red triangle, and blue squares represent *p* values of Bovine50K genotypes, experimentally validated *BovineHD0800025437* genotype, and the genotypes of candidate causative variations in–log_10_ scale, respectively.

A GWAS using BovineSNP50 genotypes from 1156 Japanese Black steers also detected *CW-3* [7]. The QTL was represented by a haplotype consisting of two single nucleotide polymorphisms (SNPs) but not by any single SNP [7]. To explore an SNP marker tagging the QTL, the BovineSNP50 genotypes were imputed to BovineHD genotypes using 651 steers as a reference population, followed by an examination for association. Twenty-two SNPs between 85.7 and 85.8 Mb were strongly associated with carcass weight (*p* < 3.1 × 10^–15^), of which the genotype of *BovineHD0800025437* was experimentally validated to show 99.2% concordance (18 inconsistent alleles among 2312 alleles). Inclusion of *BovineHD0800025437* genotype in the statistical model as a covariate resulted in the loss of all significant associations on BTA 8, indicating that the SNP is in strong linkage disequilibrium with the causative variation for *CW-3* (Fig 2A).

To further refine the *CW-3* QTL region, a haplotype-based association analysis was performed using imputed BovineHD genotypes from the GWAS population. First, the 11-Mb *CW-3* region was scanned for association with carcass weight by an approximately 1-Mb *Q* haplotype in a half-length sliding window. The most associated region, between 82.8 and 88 Mb, was then scanned by an approximately 500-kb-long window of the *Q* haplotype (Fig 2B). The results narrowed the *CW-3* region to a 3.3-Mb interval between 83.7 and 87.0 Mb (Fig 2B).

### Recessive skeletal dysplasia was mapped on 2.2-Mb *CW-3 Q* haplotype

**Fig 3:**
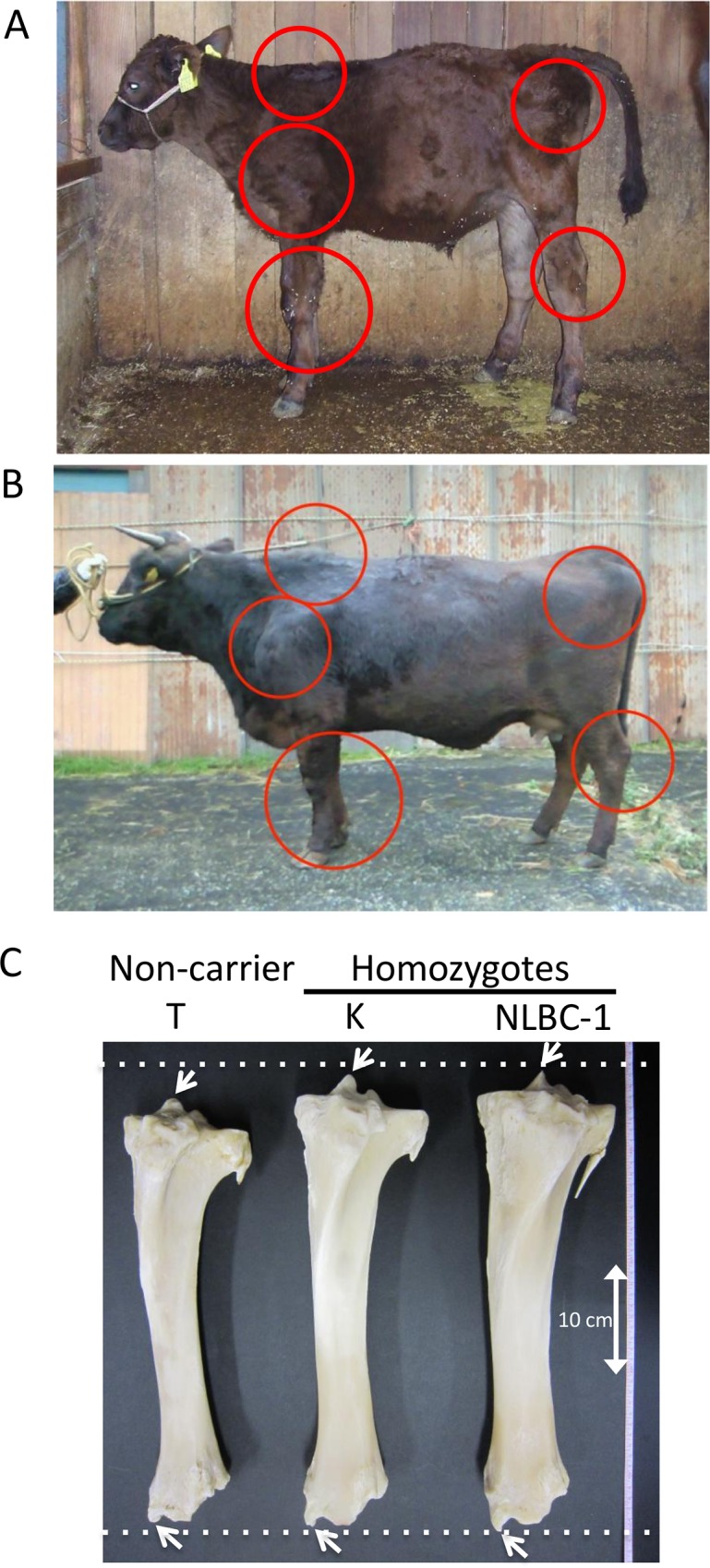
Clinical features of skeletal dysplasia. Affected calf (A) and cow (B) are presented. Red circles show the symptoms of thin neck, extended shoulder, thick joints of the limbs, and hip bone-enlargement. (C) Tibias from cows with FGD3 Cys-171 homozygous (NLBC-1 and K) and a non-carrier control (T) cows. They were 7.1–8.3 years old. Withers height (WH), shank circumference (SC), tibia length (TL), and circumference of tibia shaft (CT) are as follows: NLBC-1, 135.6 cm (WH), 20.4 cm (SC), 38.8 cm (TL), 13.6 cm (CT); K, 140 cm (WH), 18.5 cm (SC), 38.4 cm (TL), 13.0 cm (CT); T, 128.8 cm (WH), 17.8 cm (SC), 36.6 cm (TL), 12.0 cm (CT). The arrows indicate medial malleolus and intercondylar eminence. Dashed lines were drawn to facilitate observation.

**Fig 4:**
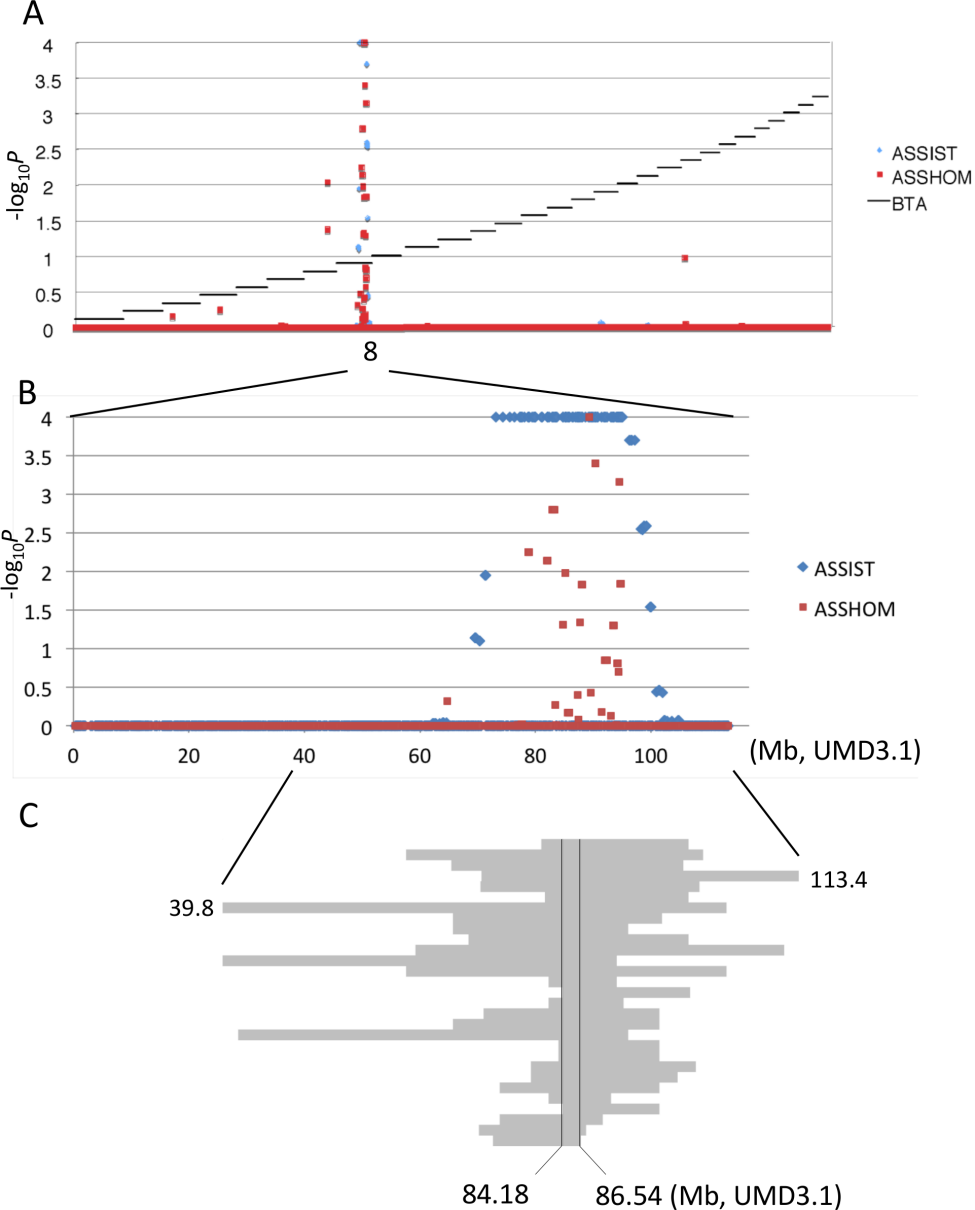
Mapping of homozygosity and autozygosity for skeletal dysplasia. (A) The results of the genome-wide homozygosity and autozygosity mapping of skeletal dysplasia using 14 affected and 34 normal animals from three families. Black horizontal bars mark the limits between the 29 autosomes. ASSHOM and ASSIST were used for mapping [14]. (B) BTA8 (C) Schematic representation of homozygous regions on BTA8 in 29 affected animals.

**Fig 5:**
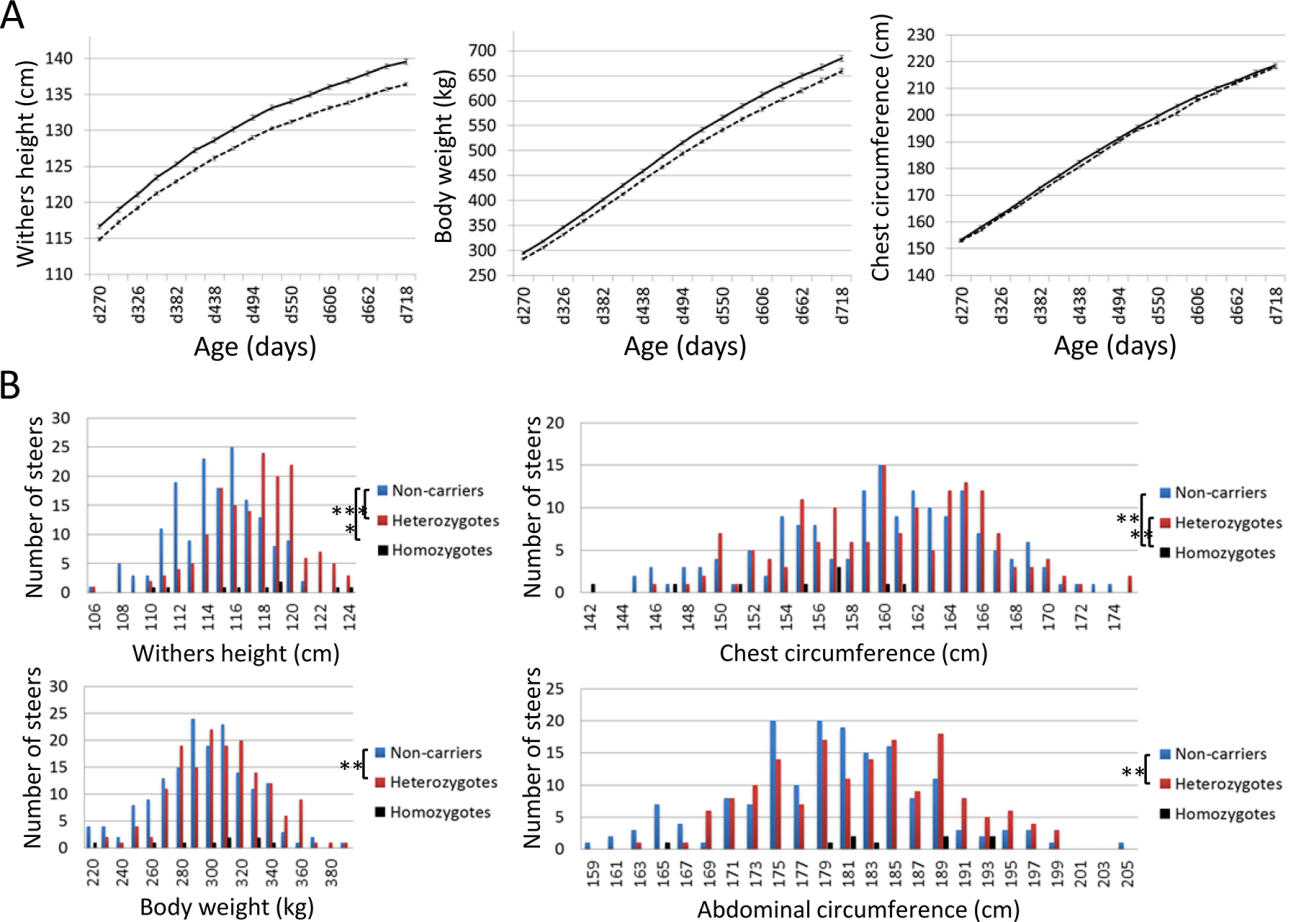
Effects of *CW-3 Q* or risk allele on skeletal measurements. (A) Growth curves of heterozygous (n = 98, solid line) and homozygous *q*-steers (n = 165, broken line). The non-synonymous SNP in *PTPDC1* was used as a marker for *CW-3*. The average and standard error (S.E.) are shown. Left, withers height; middle, body weight; right, chest circumference. (B) Distribution of skeletal measurements of calves with respective genotypes. Offspring steers from Sire R were genotyped with non-synonymous SNPs encoding His-171-Cys in FGD3. The number of non-carrier, carrier, and risk allele-homozygous animals were 165, 159, and 9, respectively. Upper left, withers height; upper right, chest circumference; lower left, body weight; lower right, abdominal circumference. *, *p* < 0.05; **, *p* < 0.01; ***, *p* < 0.001.

Skeletal dysplasia, characterized by joint- and/or hip bone-enlargement, has been known in a specific lineage of Japanese Black cattle (Fig 3A and 3B). The disease causes economic damage to farmers because affected animals are bony and not fattened well. Since affected animals were produced from Sire II and its related sires, P and Q (Fig 1A), 14 affected and 34 control animals from the three families were genotyped with the BovineSNP50 Genotyping BeadChip. Genotypes were used for homozygosity and autozygosity mapping using ASSHOM and ASSIST programs, respectively [14]. Both programs showed genome-wide significant signals on BTA8 (*p* = < 10^–4^) (Fig 4A), and the plot of *p*-values on BTA8 (Fig 4B) was similar to that of the GWAS for carcass weight [7] (Fig 2). The risk haplotype was on the same chromosome as the *CW-3 Q* haplotype in Sire II. Another 22 affected animals were collected from various sires and genotyped with BovineSNP50. Of 36 affected animals, 29 (80.6%) shared a homozygous 2.36-Mb risk haplotype between *ARS-BFGL-NGS-108358* (84.18 Mb) and *BTA-52694-no-rs* (86.54 Mb) (Fig 4C). Of the remaining seven affected animals, two did not possess the risk haplotype and five were heterozygous along BTA8. The initial mapping contained three of the seven animals, while no significant regions were detected when the 7 affected and 34 control animals were subjected to ASSHOM (*p >* 0.7) and ASSIST programs (*p >* 0.11). These seven animals may be erroneously diagnosed, because the disease phenotype is highly variable and the conditions are quantitative rather than qualitative as described below (Fig 5B and S2 Table).

The 2.36-Mb risk haplotype for skeletal dysplasia was encompassed by the 3.3-Mb *Q* haplotype of *CW-3*, indicating they are closely linked (Fig 1B). Although the *IARS* gene (85.3 Mb), whose missense mutation has been identified as the cause of hereditary perinatal weak calf syndrome in Japanese Black cattle [15], is located within the 2.36-Mb region, the risk haplotypes were different for the two diseases.

### Targeted resequencing identified non-synonymous mutations in *FGD3* and *PTPDC1*

To search for candidate causative variations for skeletal dysplasia and/or *CW-3*, three sires segregating the *CW-3* QTL (Sires II, V, and VII), three *non-Q* homozygous sires (Sires D, I, and J), and a *Q*-homozygous steer (Off-23) were subjected to targeted resequencing as described previously [7]. The 3.3-Mb *CW-3* region contained 910 candidate QTNs (858 SNPs and 52 indels), including four non-synonymous and five synonymous SNPs. Coding regions that were not covered by targeted resequencing were read by Sanger sequencing, where four synonymous SNPs were detected (S3 Table). Non-synonymous SNPs were located in *FGD3* (*FYVE*, *RhoGEF*, and *PH domain containing 3*) (85.8 Mb) and *PTPDC1* (*protein tyrosine phosphatase domain-containing protein 1*) (86.8 Mb), and synonymous SNPs were located in *BICD2* (*protein bicaudal D homolog 2*) (85.7 Mb) and *FGD3* (S4 Table). All non-synonymous SNPs showed strong association with carcass weight (Fig 2C). The three non-synonymous SNPs in *FGD3* showed complete linkage disequilibrium, while the linkage disequilibrium coefficient (*r* ^2^) between the non-synonymous SNP in *PTPDC1* and those in *FGD3* was 0.907 in the GWAS population.

Using the non-synonymous SNP in *PTPDC1* as a *CW-3* marker, growth curves were compared between heterozygous (n = 98) and homozygous *q*-steers (n = 165), indicating a highly significant effect of the *Q* allele on withers height (*p* < 1 × 10^–4^) and body weight (*p* < 0.003) during all test periods, while the effect on chest circumference was significant only at day 382 (*p* = 0.039) and day 438 (*p* = 0.020) (Fig 5A). The results show that *CW-3* primarily affects the stature as previously reported for other carcass weight QTL, *PLAG1*/*CW-1* [3,16] and *CW-2* [17].

*BICD2* has been recently reported as the causative gene for spinal muscular atrophy in human [18–20]. The disease is characterized by lower-limb-predominant weakness [18–20], which is different from skeletal dysplasia (Fig 3A and 3B) and *CW-3* (Fig 5A). Therefore we eliminated *BICD2* from candidate genes and did not further examine synonymous SNPs in *BICD2*.

### *PTPDC1* is excluded from candidate genes for *CW-3*

*PTPDC1* was previously reported as one of 180 loci for adult human height [21], while it constitutes the 237^th^ locus with *C9orf3*, *PTCH1* and *HABP4* in the most recent analysis that identified 697 variants clustered in 423 loci affecting adult human height [4]. The locus was defined as one or multiple jointly associated SNPs located within a 1-Mb window and in the 237^th^ locus the majority of signals cluster in and around *PTCH1* [4] that is known to relate to body size [22]. In bovine, there is an intra-chromosomal rearrangement between *PTPDC1* (86.8 Mb) and other three genes (82.6–84.6 Mb).

To examine a possibility of *PTPDC1* as a responsible gene for *CW-3*, we first searched for the sires that have a recombination between *FGD3* and *PTPDC1*. Sire B [23] was found to be heterozygous for the non-synonymous SNP in *PTPDC1* but had homozygous *q* alleles for the non-synonymous SNPs in FGD3 (Fig 1B). An IBD analysis indicated that Sire B is heterozygous *Q*/non-*Q* between *MS087* (86.0 Mb) and *MS091* (87.5 Mb) of the *CW-3* QTL interval (Fig 1B), while a QTL for carcass weight was not detected in a previous QTL mapping study using 328 progeny of this sire [23](S1 Fig). These data strongly suggest that *CW-3* maps centromeric to *MS087* (Fig 1B). Consistent with the results, *Ptpdc1*-deficient mice did not show any differences in body weight (at 4–12 weeks of age) or body length (at 12 weeks of age) (S4 Table; 9–21 mice in respective genotypes), indicating that *PTPDC1* is neither a height gene nor the cause for *CW-3*. Although *Ptpdc1* was highly expressed in testes (S2 Fig), homozygous *Ptpdc1*-deficient mice were fertile (five *Ptpdc1* ^-/-^ male mice when mated with *C57BL/6J* females, of which four produced offspring).

The stronger association of the non-synonymous SNP in *PTPDC1* than non-synonymous SNPs in *FGD3* that are located centromeric to *MS087* (Fig 2C) is probably due to sampling bias by chance. Only 19 animals had a haplotype with recombination between *FGD3* and *PTPDC1*, of which five had a *q-Q* and 14 had *Q-q* haplotypes.

As for skeletal dysplasia, an affected animal has never been observed in the offspring from Sire B, suggesting that the causal mutation is located within the 1.8-Mb interval between 84.2 and 86.0 Mb (Fig 1B).

### A non-synonymous variant of FGD3 causes reduced GEF activity

**Fig 6:**
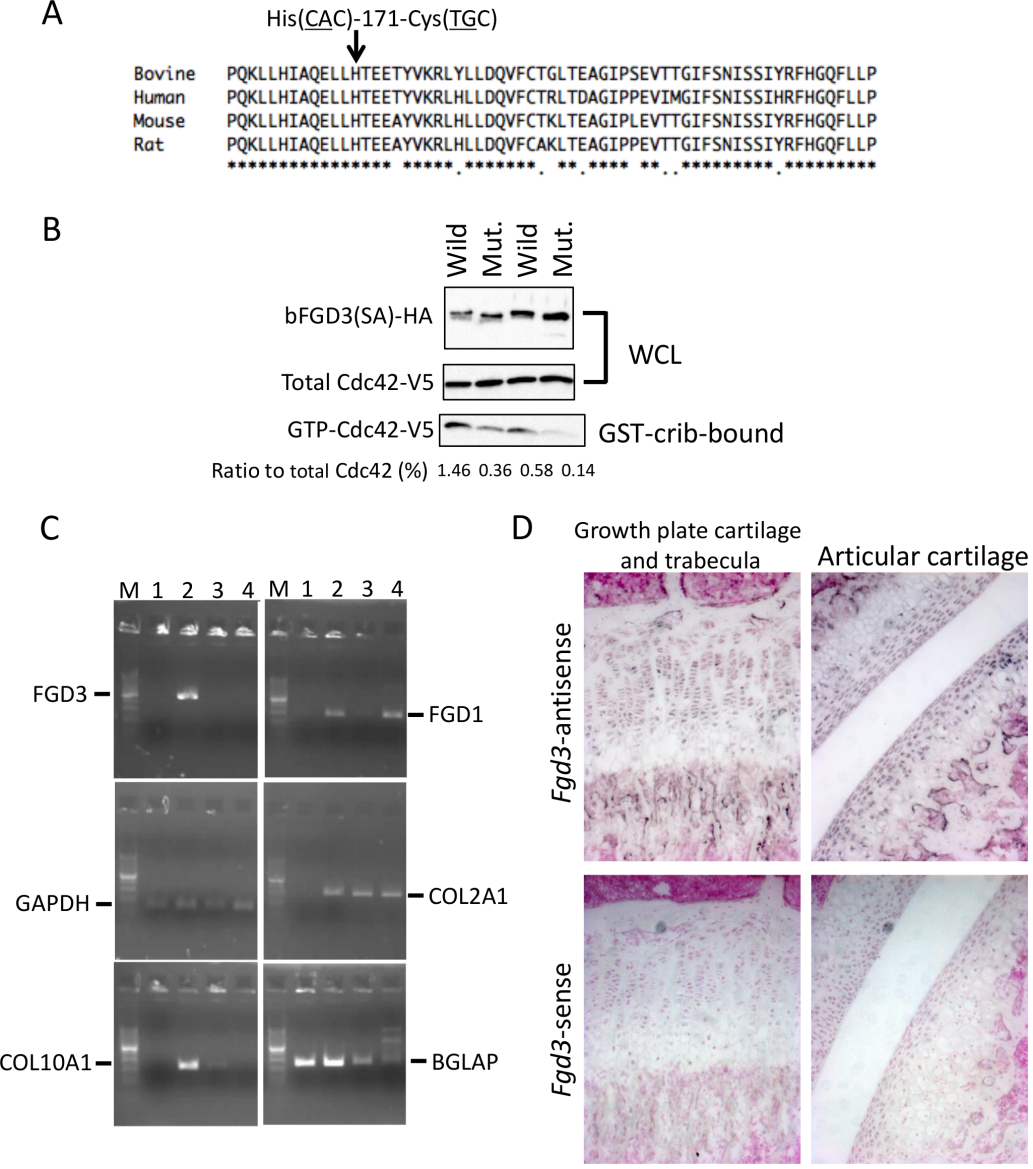
Amino acid sequence alignment around His-171, GEF activity, and gene expression of FGD3. (A) His-171 of bovine FGD3 and the flanking region is well conserved among mammalian species. The RhoGEF domain corresponds to the region from His-164 to Ala-344 of bovine FGD3. (B) NIH3T3 cells were transfected with the bicistronic expression plasmid encoding HA-tagged wild or mutant bovine FGD3(SA) and V5-tagged Cdc42. FGD3(SA) denotes FGD3 with two serines in the destruction motif (Ser-83 and Ser-87 of bovine FGD3) replaced by alanines [24]. After 48 hours, cell extracts were prepared and submitted to the GST–CRIB pull-down assay. A portion of cell extracts and the pull-down products were subjected to SDS-PAGE followed by immunoblotting to detect Cdc42-V5 and FGD3(SA)-HA. The experiment was repeated two more times, and the results are shown in S4 Fig. (C) RNA was extracted using RNAiso Plus (Takara) from tissues of a 1-month-old Holstein calf. Primary chondrocytes were prepared from the ear cartilage and cultured as micromass. Five days later, RNA was extracted from the micromass using an RNeasy kit (QIAGEN, Valencia, CA, USA). RT-PCR was performed using a standard method and PCR primers given in S8 Table. PCR products were resolved in a 2% agarose gel. Lane M, 100-bp DNA ladder; lane 1, bone marrow just under the growth plate of a femur; lane 2, growth plate cartilage of the femur; lane 3, ear cartilage; lane 4, micromass culture from the ear cartilage. (D) *In situ* hybridization of *Fgd3* on the femur of a 3-week-old C57BL/6 mouse. Sense and antisense RNA probes correspond to nt. 129–956 of the mouse *Fgd3* cDNA (NM_015759).

*FGD3* located at 85.8 Mb is the only gene that has non-synonymous SNPs within the critical interval for skeletal dysplasia (Figs 1B and 4C). The encoding protein functions as a guanine nucleotide exchange factor (GEF) for Cdc42 [24]. The five synonymous SNPs in *FGD3* (S3 Table) were verified not to affect splicing (S3 Fig), and they appeared to have no substantial effect on translation efficiency (Codon usage database, http://www.kazusa.or.jp/codon/). In contrast, the three non-synonymous SNPs in *FDG3* caused loss of the start codon (ATG to ACG) and an amino acid substitution from His-171 (CAC) to Cys (TGC). Since the Kozak consensus sequence is present at the second Met of the 17th amino acid residue (S4 Fig), the *FGD3* variant probably produces an N-terminal 16 amino acid-truncated protein. The His-171-Cys substitution is located in the GEF domain, where a His residue is conserved among mammalian species (Fig 6A). In order to examine GEF activity of the wild and mutant FGD3 proteins, a pull-down assay was performed using a GST-PAK Cdc42/Rac interacting binding (CRIB) fusion protein. The wild and mutant FGD3 proteins were expressed as FGD3(SA) in which two Ser residues of the DSGIDS motif were altered to Ala residues to protect them from proteasomal degradation [24]. As presented in Fig 6B and S4 Fig, the mutant protein showed reduced GEF activity.

### FGD3 expresses in growth plate cartilage

Both skeletal dysplasia (Fig 3) and *CW-3* (Fig 5A) are related to bone development. As a primary candidate gene, expression of *FGD3* was examined in bone tissues. The results of the RT-PCR showed that bovine *FGD3* was strongly expressed in the growth plate cartilage from a Holstein femur at 1 month of age but not expressed in ear cartilage or in bone marrow just under the growth plate (Fig 6C). *GAPDH* was used as a positive control and *COL2A1*, *COL10A1*, and *BGLAP* were used as markers for chondrocytes, hypertrophic chondrocytes, and osteoblasts, respectively. *FGD1* is the causative gene for human faciogenital dysplasia that is characterized by short stature, facial abnormalities, and skeletal and genital anomalies [25]. *FGD1* was induced in micromass-cultured chondrocytes from ear cartilage, while *FGD3* was not (Fig 6C).

Tibias were obtained from two FGD3 Cys-171 homozygous cows (NLBC-1 and K in Table 1) and a non-carrier control cow (T in Table 1). Tibias from the homozygotes were longer and thicker than that of the non-carrier cow (Fig 3C). In addition, medial malleolus and intercondylar eminence are more prominent in homozygotes. Overgrowth of the articular cartilage may be the cause of joint-enlargement, a phenotypic character of affected animals. To examine *FGD3* gene expression precisely, *in situ* hybridization was carried out using a mouse femur. *Fgd3* was expressed in osteoblasts and proliferating chondrocytes of the growth plate and articular cartilages (Fig 6D), which were generally consistent with the regions where bone abnormalities were observed in the homozygous cows (Fig 3C).

**Table 1:** Deviations of the skeletal measurements of the FGD3 Cys-171 homozygotes and a non-carrier cow from normal growth curves.

ID	Sex^1)^	Months of age	Chest depth^2)^	Chest width^2)^	Thurl width^2)^	Pin-bone width^2)^	Characte-ristics^3)^	Comments
48	M	3.0			-2.9	-2.2	A	Necrotizing suppurative bronchopneumonia
A-8	M	3.2	1.8	-2.5	2.8	3.0	B	
A-6	M	5.7	1.6	-0.3	3.9	3.1	B	
49	M	6.6	-2.1	-2.1	-1.4	0.0	A	Thoracic curvature
A-9	M	6.7	-4.5	-4.6	-2.6	-3.4	A	
A-3	M	7.3	2.5	0.2	2.7	-0.4	B, C	
S-16	M	8.2	-0.2	-1.4	0.4	0.7	B	Spinal curvature
S-13	M	8.3	1.8	-2.4	3.3	-0.4	B, C	
S-12	M	8.5	-0.1	-1.2	1.7	-1.6	B, C	
S-8	M	10.0	-2.1	0.7	1.5	-2.6	A, C	
A-2	M	10.8	-0.6	-4.3	0.9	-2.4	A, B, C	
A-14	M	10.8	2.1	-2.2	1.4	0.0	B, C	
A-12	M	15.1	-0.8	-0.6	2.8	-2.8	C	
A-11	M	26.2	0.3	-2.4	2.1	-1.7	B, C	
SH	M	26.7	1.9	0.3	3.3	0.1	B, C	
A-10	F	2.5	2.9	-0.7	5.0	0.1	B, C	
47	F	5.4	-1.7	-0.9	1.2	0.4	A	Diagnosed as poor development in a livestock hygiene sevice center
A-15	F	21.9	2.6	1.3	4.4	-1.2	B, C	
D-1	F	42.0	1.5	-0.2	2.4	2.4	B	
D-2	F	85.7	0.2	-0.5	1.9	1.0		Found by genetic testing and screening
D-3	F	70.0	1.8	0.2	4.0	2.2	B, C	Found by genetic testing and screening
NLBC-2	F	85.3	4.0	1.3	4.0	2.6	B, C	Found by genetic testing and screening
D-1	F	17.6	1.5	0.7	2.6	2.5		
NLBC-2	F	14.5	2.2	2.5	2.8	1.4	C	
NLBC-1	F	22.0	1.3	0.8	1.2	-0.4	C	
K	F	27.1	0.3		-1.9			
T	F	99.2	0.6	0.2	1.0	1.0		Non-carrier control

^1)^F, female; M, male or steer.

^2)^Deviations from normal growth curves (sigma value) are shown.

^3)^A, poor development; B, disproportionately narrow chest width; C, disproportionately wide thurl width. Each definition is described in Materials and Methods. A full table is shown in S2 Table.

### Both skeletal dysplasia and *CW-3* are explained by disproportional tall stature

To characterize the disease phenotypes, skeletal measurements were collected from risk allele-homozygous animals (Table 1 and S2 Table). Their ages and conditions were varying and spinal or thoracic curvature was rare. To compare the measurements from the animals of different ages, deviations from normal growth curves were calculated. Six (25%) of the 24 animals were recognized as having poor development. These animals were confirmed to be free from the *IARS*-risk haplotype or Val-79-Leu mutation of *IARS* [15]. Of the remaining 18, 16 showed disproportionately narrow ratio of chest width to chest depth and 12 showed disproportionately wide ratio of thurl width to pin-bone width. Poor development and narrow chest width are correlated with economic loss to farmers. Although the phenotypes of skeletal dysplasia were thought to be congenital, some exceptions were found in this study. Four cows that were previously not recognized as affected at younger ages (14.5–22 months of age), showed a disproportion between chest width and chest depth at later ages (3.5–7.8 years of age) (Table 1). In contrast, a proportion of thurl width to pin-bone width appeared unchanged irrespective of age (Table 1).

To further examine the skeletal measurements of the risk-allele homozygotes, 332 offspring steers from the FGD3 His-171-Cys heterozygous sire, Sire R (Fig 1A), were genotyped. Imputed SNP genotype showed that Sire R shared the *CW-3 Q* haplotype spanning the 11 Mb-interval from 76.5 to 87.5 Mb. The heterozygous calves were significantly taller (*p* < 0.001) and heavier (*p* < 0.01) than the non-carrier calves, but the chest circumferences were not different between these two groups (*p* = 0.34) (Fig 5B). The data were consistent with the results from the progeny tests (Fig 5A), suggesting that the risk allele causes disproportional tall stature. The hypothesis was verified by the measurements of the risk allele-homozygous calves: they were taller (*p* < 0.05) but had smaller chest circumference (*p* < 0.01) than the non-carrier calves (Fig 5B), indicating that disproportional tall stature is caused in an allele-dosage dependent manner. Body weight of the calves with homozygous risk alleles varied widely and was not significantly different from that of either the carrier or non-carrier calves (Fig 5B). The smallest calf with homozygous risk alleles showed extremely small chest circumference that was classified as poor development, which may represent a severe case of the disease (Table 1). The carcass data at slaughter confirmed an increase in carcass weight as *CW-3* in the carrier status (*p* < 0.001, Table 2). The carcass yield estimates of heterozygous steers were lower than those of non-carrier steers (*p* = 0.0013), suggesting that the risk allele increases a ratio of bone to carcass weight. Carcass data of the risk allele homozygous animals could be traced for six of the nine animals. They were worse than those from heterozygous and non-carrier steers (S5 Table).

**Table 2:** Effect of the risk allele on carcass traits.

	Average	SD	*p*-value^1)^	Allele substitution effect	SE	% variance explained
Cold carcass weight (kg)	495.9	65.3	< 0.001	33.9	9.4	12.0
Longissimus muscle area (cm2)	64.0	10.7	n.s.			
Rib thickness (cm)	8.0	0.94	n.s.			
Subcutaneous fat thickness (cm)	2.6	0.78	n.s.			
Beef marbling standard	8.2	2.0	n.s.			
Carcass yield estimate (%)	74.5	1.5	< 0.01	-0.72	0.22	5.8

^1)^A linear regression analysis was performed using non-carrier (n = 86) and carrier offspring steers (n = 85) from Sire R. Slaughter age was included in the model as a covariate. n.s., not significant.

These results indicate that the risk allele causes disproportional tall stature in an additive manner. *CW-3* is a result of an increase in height with unchanged width, while the homozygotes display skeletal dysplasia including poor development and disproportionately narrow chest width.

## Discussion

This study revealed that a QTL for carcass weight, *CW-3*, is closely linked with recessive skeletal dysplasia. Skeletal measurements of calves revealed that the risk allele (*CW-3 Q* allele) causes disproportional tall stature in an allele dosage-dependent manner, suggesting that skeletal dysplasia and *CW-3* are attributed to the same mutation. Only *FGD3* has a non-synonymous coding variant within the critical region for skeletal dysplasia and the mutant protein showed reduced GEF activity, strongly suggesting that a mutation in *FGD3* is causative.

The mammalian FGD gene family consists of six members. Mutations in human *FGD1* cause faciogenital dysplasia affecting multiple skeletal structures including short stature [26]. FGD6 was recently shown to regulate endosomal membrane recycling in osteoclasts [27]. FGD3, similarly to other FGD proteins, functions as a GEF for Cdc42 [24], however the specific role of FGD3 has been unknown. Lacroix et al. [28] reported that FGD3 is overexpressed in follicular thyroid tumors with *PAX8*-*PPARγ1* rearrangement and localized at the lateral membrane but not at the basal or apical membranes. Because *FGD3* is expressed in the growth plate cartilage (Fig 6C and 6D), reduced GEF activity of FGD3 might reduce the active Cdc42 at the lateral membrane of chondrocytes, which may lead to columnar disorganization of chondrocytes seen in both limb bud mesenchyme-specific inactivated Cdc42 (*Cdc42^fl/fl^; Prx1-Cre*) mice [29] and chondrocyte-specific inactivated Cdc42 (*Cdc42^fl/fl^; Col2-Cre*) mice [30]. These cell type-specific inactivated Cdc42 mice indicate the essential role of Cdc42 in cartilage development during endochondral bone formation. However, the role of Cdc42 in postnatal limb skeletal growth and growth plate organization and function remains unclear because most of *Cdc42^fl/fl^; Prx1-Cre* mice die within a few days [29] and nearly all *Cdc42^fl/fl^; Col2-Cre* mice die within 1 day after birth [30]. Furthermore, another GEF for Cdc42, such as *Fgd1*, is also expressed in growth plate chondrocytes [26], which may account for shorter limbs and bodies in *Cdc42^fl/fl^; Prx1-Cre* and *Cdc42^fl/fl^; Col2-Cre* neonate mice [29,30]. Therefore, generation and analyses of *Fgd3*-deficient mice may be crucial to verify the causality of *Fgd3* for skeletal dysplasia.

Disproportional tall stature is caused by activation of the C-type natriuretic peptide (NPPC)/NPR2 pathway. C-type natriuretic peptide binds to NPR2 in growth plate chondrocytes, which functions as a guanylyl cyclase to increase intracellular cGMP level [31]. The increase in cGMP level further activates cGMP-dependent protein kinase II and seems to promote the accumulation of extracellular matrix in the growth plate [32]. A spontaneous loss-of-function mutation of mouse *Npr2* causes severe disproportionate dwarfism [33], while a gain-of-function mutation of *NPR2* producing excessive cGMP causes human overgrowth disorder [34]. The transgenic mouse model expressing the gain-of-function mutant Npr2 in chondrocytes exhibits bone deformities, which, depending on the expression levels of the transgene, include elongation of the spine with severe kyphosis or elongated spinal and tail vertebrae and phalanges with mild kyphosis [34]. NPR3 functions as a natriuretic peptide clearance receptor and the NPR3 inactivated mice also show a disproportionate tall stature [35]. The diverse phenotypes of the mice expressing the gain-of-function mutant Npr2 resemble various conditions of bovine skeletal dysplasia. Thus, a reduced activity of bovine FGD3 may induce growth plate disorganization, which may in turn lead to activation of the NPPC/NPR2 pathway. NPR2 is also located on BTA8 but its genomic position (60.4 Mb) is apart from the critical region for skeletal dysplasia (Fig 4C).

Fasquelle et al. [13] showed that a frame-shift mutation in *MRC2* causing the recessive Crooked Tail Syndrome gives desired characteristics such as increased muscularity in the carrier status in Belgian Blue cattle. Likewise, *CW-3 Q* allele increases carcass weight in the heterozygous state. Because *CW-3* heterozygous bulls tend to be preferentially selected due to enhanced longitudinal growth, marker-assisted breeding will be useful to avoid an increased frequency of skeletal dysplasia.

## Materials and Methods

### Ethics statement

This research was approved by the National Livestock Breeding Center for Animal Research (H26-5) and conducted in accordance with the Institutional Animal Care and Use Committee Guidelines from National Livestock Breeding Center.

### QTL mapping

QTL analyses were performed with the interval mapping method using a linear regression model for half-sib families, as described previously [7]. Slaughter year and age (day) were included as fixed effects in a model. Marker locations were obtained from the Shirakawa-USDA linkage map [36].

### Development and genotyping of microsatellite and SNP markers

Genomic sequences were examined to identify microsatellites. Primers targeting microsatellites or SNPs were designed using Primer 3 (http://bioinfo.ut.ee/primer3/) [37]. The UMD3.1 assembly was used for genomic positions. Genotyping of microsatellites was performed using polymerase chain reaction (PCR) with a fluorescently labeled reverse primer, followed by electrophoresis using an ABI 3730 DNA analyzer (Applied Biosystems, Foster City, CA, USA) and analysis using GeneMapper software (Applied Biosystems). The sires and their offspring were genotyped to determine the phase of the sires’ chromosomes. For genotyping of SNPs, direct sequencing of the PCR products was performed using BigDye Terminator v.3.1 Cycle Sequencing Kit (Applied Biosystems), followed by electrophoresis using an ABI 3730 DNA analyzer. The primer sequences are shown in S6 Table.

### Imputation and haplotype-based association analysis

The GWAS population consisted of 1156 Japanese Black steers whose carcass weight had higher ratios of both extremes than the ratio observed in collected samples (> 27,500) but was normally distributed [7]. The BovineSNP50 (Illumina, San Diego, CA, USA) genotypes on BTA8 of the GWAS population were imputed to BovineHD (Illumina) genotypes using Beagle 3.3.2. [38]. The BovineHD genotypes of 651 Japanese Black steers that passed our quality-filter [7] were used as a reference population. Association of imputed genotypes with carcass weight was examined using EMMAX software [39]. The IBS matrix was made using BovineSNP50 genotypes as previously described [7]. To examine imputation accuracy, an SNP, *BovineHD0800025437*, was genotyped for the 1156 steers by direct sequencing of the PCR products (S6 Table).

The sires segregating *CW-3* (Sires I-VII), their common ancestor (Sire X), and an offspring from Sire VI (Off-6) were genotyped with BovineHD Beadchips (Illumina), followed by imputation as described above. Since imputed genotypes were obtained as phased haplotypes, the *CW-3 Q* haplotype consisting of BovineHD SNPs was obtained as a shared haplotype among the sires and confirmed by the Off-6 genotype that possessed homozygous *Q* alleles between 77.8 and 100.9 Mb.

For a haplotype-based association analysis, one out of every nine SNPs was taken from the phased BovineHD genotypes of the GWAS population to reduce the number of SNPs that constitute haplotypes. The average interval of the SNPs was 50 kb. Haplotypes consisting of 20 contiguous SNPs, whose length was estimated to approximately 1 Mb, were divided into *Q* or non-*Q* haplotypes. Association of *Q* haplotypes with carcass weight was examined with a sliding window of 10 SNPs using EMMAX software [39] as described above. For an analysis of approximately 500-kb haplotypes, the first and fifth of every nine SNPs were used.

### Mapping of skeletal dysplasia

The initial mapping was done with 14 affected and 34 control animals from three families: 5 affected and 10 control offspring from Sire II, 5 affected and 10 control offspring from Sire P, and 4 affected and 12 control offspring from Sire Q. Sires P and Q were also used as control animals. Both affected and control animals included males and females. They were genotyped with BovineSNP50 Beadchips and submitted to homozygosity and autozygosity mapping using ASSHOM and ASSIST programs, respectively [14]. Further, 22 affected animals were collected from various groups of sires and genotyped with BovineSNP50 Beadchips. A total of 36 affected animals were used to search for a shared homozygous region on BTA8.

### Targeted resequencing

Three sires segregating the QTL (Sires II, V, and VII), three *non-Q*-homozygous sires (Sires D, I, and J) [7], and a *Q*-homozygous offspring from Sire VI (Off-23) were subjected to sequence capture (NimbleGen custom array), followed by resequencing (Illumina GAIIx, 40-bp paired-end run). This experiment was performed in parallel with the targeted resequencing of the *CW-1* region [7]. *Non-Q*-homozygous sires were defined as those that harbored homozygous *q* alleles at *BovineHD0800025437* and did not segregate a carcass weight QTL on BTA 8 in the half-sib analyses using more than 236 offspring per family.

Targeted region included an 11-Mb interval of the *CW-3* region from 76.5 to 87.5 Mb on UMD3.0. Obtained putative sequence variations were filtered by: (1) heterozygous in the three sires segregating *CW-3* QTL, (2) homozygous in the three *non-Q*- and *Q*-homozygous animals, and (3) opposite alleles between the *Q*- and *non-Q*-homozygous animals.

Coverage of the coding regions in the 3.3 Mb-interval between 83.7 and 87.0 Mb was checked in each animal. The regions with fewer than four reads in either of the animals were subjected to Sanger sequencing (S3 Table).

### Effects of *CW-3 Q* or the risk allele on growth metrics

Two hundred and sixty-four steers from 35 sires, which were used for progeny tests at Shimane Prefectural Livestock Technology Center from 2002 to 2011, were genotyped for non-synonymous SNP in *PTPDC1* (S3 Table; *bPTPDC1_e6_FV* in S6 Table). There was only one steer that had homozygous *Q* alleles (= G) and was excluded from the analysis. The measurements of withers height, body weight, and chest circumference were interpolated by cubic spline at 4-week-intervals starting at 270 days of age. Significance of the difference between heterozygous and homozygous *q*-steers was tested using Student’s *t*-test.

Skeletal measurements of calves, withers height, body weight, and chest and abdominal circumferences, were collected at the stock market in Miyagi Prefecture from October in 2008 to March in 2009. Hair roots were collected for DNA samples. Of those, 333 offspring steers from Sire R were genotyped for the SNPs encoding His-171-Cys in FGD3 (S3 Table; *FGD3_e2_HC* in S6 Table). Sire R was heterozygous for the SNPs. The age of the calves was 295 ± 21 days. The phenotypic values were compared between genotypes using ANOVA. Approximately half of the genotyped animals could be traced for the carcass data at time of slaughter. Because the carcass data of the FGD3 Cys-171 homozygotes were significantly worse than those of other genotypes, they were excluded from the subsequent linear regression analysis. Association of the risk allele with carcass traits was examined using a linear regression model including slaughter age as a covariate. Sire R was genotyped using Axiom Genome-Wide BOS1 Array Plate (Affymetrix, Santa Clara, CA, USA), followed by imputation to Bovine HD genotype as described above.

### Growth of *Ptpdc1*-deficient mice

Heterozygous *Ptpdc1*-deficient mice (TF0596) were obtained from Taconic Knockout Mouse Repository (Taconic Biosciences, Inc., Hudson, NY, USA). The targeted allele was deleted from exon 4 to exon 7 of the *Ptpdc1* gene and replaced by *LacZ* and *Neo* genes. The heterozygous males were backcrossed into *C57BL/6J* females (CLEA Japan, Tokyo, Japan) four times and the resultant heterozygous males and females were crossed to obtain littermates with respective genotypes. Genotyping was performed by PCR analysis. The PCR reaction was done using TAKARA LA Taq and GC buffer I (Takara, Tokyo, Japan) in a single reaction tube containing the following three primers: Neo_new, 5’-TCGCCTTCTTGACGAGTTCT-3’; TF0596-wild, 5’-CCCTGTAGCCCTCTGAACTG-3’; TF0596-31, 5’-GGGCAGGTTCTGTTTCTCTG-3’. The primer set Neo_new and TF0596-31 amplifies 247 bp of the targeted allele, while the primer set TF0596-wild and TF0596-31 amplifies 450 bp of the wild allele. Body weight of calves at the age of 4 to 12 weeks and body length at 12 weeks were compared among genotypes by ANOVA.

### cDNA construction and plasmids

cDNAs were amplified by PCR using PrimeSTAR GXL polymerase (Takara). The PCR primers contained a 15-bp sequence of the 5’-end of the cloning vector or of another primer sequence. The primer sequences are shown in S7 Table. Cloning reactions were performed using In-fusion HD cloning kit (Clontech Laboratories, Inc., Mountain View, CA, USA). All of the amplified sequences were verified by dideoxy sequencing using BigDye Terminator v.3.1 Cycle Sequencing Kit (Applied Biosystems).

Briefly, a cDNA encoding full-length bovine FGD3 was amplified by PCR from the bovine fetus kidney cDNA library [40] and inserted into the pCAGGS expression vector [41] with the addition of the C-terminal HA tag. Ser-83 and Ser-87 of the resultant bFGD3-HA cDNA were replaced with alanines and inserted into the pIRES2-EGFP vector (Clontech). The resulting plasmid was termed bFGD3SA-HA/pIRES2-EGFP. His-171-Cys substitution was created from bFGD3-HA cDNA. The SNP at the initial Met was introduced together with replacing the Ser-83 and Ser-87 residues with alanines and inserted into the pIRES2-EGFP vector; the resulting plasmid was termed bFGD3SA-2ndMCys-HA/pIRES2-EGFP. Myc-tagged Cdc42 [24] was cloned into pcDNA3.1/V5-His TOPO TA vector (Life Technologies, Carlsbad, CA, USA) to add V5-His tag at the C-terminus. Then, the BstXI-NotI fragments encoding EGFP of bFGD3SA-HA/pIRES2-EGFP and bFGD3SA-2ndMCys-HA/pIRES2-EGFP were replaced with Myc-Cdc42-V5-His, resulting in bFGD3SA-HA/pIRES2-Cdc42V5His and bFGD3SA-2ndMCys-HA/pIRES2-Cdc42V5His, respectively. These plasmids were designed to bicistronically express wild or mutant FGD3(SA) and Cdc42.

### GST-CRIB pull-down assay

NIH3T3 cells were transfected with bFGD3SA-HA/pIRES2-Cdc42V5His or bFGD3SA-2ndMCys-HA/pIRES2-Cdc42V5His using Lipofectamine 2000 (Life Technologies) according to the manufacturer’s instructions, followed by the incubation for 48h. The cell lysis and pull-down assays were performed as described previously [24]. HA and V5 tags were detected using Anti-HA-tag HRP-DirectT (MBL International Corporation, Woburn, MA, USA) and Anti-V5-HRP (Life Technologies) with Amersham ECL Plus Western Blotting Detection Reagents (GE Healthcare UK Ltd., Buckinghamshire, UK), respectively. Chemiluminescence of the respective protein bands was quantified using ImageQuant LAS 4000 (GE Healthcare).

### Calculation of deviations of skeletal measurements from normal growth curves

The Japan Wagyu Register Association [42] provided normal growth curves of 10 skeletal measurements and body weight for sires and females and of withers height, chest circumference, and body weight for steers. A clear difference was observed in chest circumference between sires and steers at more than 12.5 months of age. Thus, the Japanese Black population consisting of 792 steers from a progeny testing program at the Cattle Breeding Development Institute of Kagoshima Prefecture [17] was used to obtain a normal growth curve for every measurement in steers. Growth models were used according to the Japan Wagyu Register Association [42]. Because the average age of steers at start of the progeny test was approximately 9 months, skeletal measurements of steers were divided into two groups at 10 months of age. For measurements collected from steers that were less than 10 months old, the growth curves for sires provided by The Japan Wagyu Register Association [42] were used as a standard, while the growth curves that we produced were used as a standard for measurements of steers that were more than 10 months old.

The animals with at least two measurements of less than 2σ (S.D.) were recognized as poorly developed. One exception (ID = 47), which had one measurement of less than 2σ, was defined as poorly developed because it was diagnosed as such at a livestock hygiene service center. An imbalance between chest depth and width or thurl width and pin-bone width was considered only if the difference between the two measurements was more than one σ.

## Supporting Information

S1 Fig *F*-statistic profiles for carcass or body weight on BTA 8. Summary of the quantitative trait locus (QTL) analyses is shown in S1 Table. Profiles for Sires I and II were described previously [7]. Marker locations were obtained from the Shirakawa-USDA linkage map [36]. Boxes on the x-axis indicate the 95% confidence interval of the QTL. Horizontal lines indicate the thresholds for chromosome-wise 0.1% (- --), 1% (––), and 5% (—–) significance levels.
(TIF)

S2 Fig *Ptpdc1* gene expression in mouse. (A) X-gal staining of testes from *Ptpdc1* ^+/-^ and wild-type littermates at 5-weeks of age. (B) Quantification of gene expression by real-time PCR. RNA was extracted from tibias and a testis of C57BL/6 mice. Real-time PCR was performed using TaqMan Gene Expression Assays (Applied Biosystems): *Ptpdc1*, Mm01327051_m1; *Hprt*, Mm01324427_m1. *Hprt* was used as an endogenous control. Data represent mean **±** standard deviation (S.D.) in triplicate. Bone-1 and Bone-2 were from different individuals.
(TIF)

S3 Fig Synonymous SNPs in *FGD3* did not affect splicing. RNA was extracted from testes of three heterozygous calves using RNeasy (QIAGEN). RT-PCR was performed to amplify adjacent exons of a synonymous SNP. PCR primers are shown in S8 Table. PCR products were resolved in a 2% agarose gel and confirmed not to contain an additional band (A). A representative of the sequencing profile of the PCR products is shown from (B) to (D). In the panel (B), the read was converted to show the sense strand.
(TIF)

S4 Fig N-terminal amino acid sequence of FGD3 and the GST–CRIB pull-down assay. (A) The Kozak consensus sequence is present at the first and second methionine residues of bovine FGD3, shown by underlines. (B) The other two experiments of the GST–CRIB pull-down assay.
(TIF)

S1 Table Summary of carcass or body weight quantitative trait locus (QTL) detected on BTA 8. (XLSX)

S2 Table Deviations of the skeletal measurements of affected and control animals from normal growth curves. (XLSX)

S3 Table Sanger sequencing of uncovered coding regions in the 3.3-Mb *CW-3* region. (XLSX)

S4 Table Non-synonymous and synonymous SNPs in the 3.3-Mb *CW-3* region. (XLSX)

S5 Table Body weight and length in *PTPDC1*-deficient mice. (XLSX)

S6 Table Carcass data of the offspring steers from Sire R. (XLSX)

S7 Table Marker information of microsatellites and SNPs used for identical-by-descent (IBD) analysis. (XLSX)

S8 Table Primer sequences used for RT-PCR and construction of plasmids. (XLSX)
