# Supplementary figures and images for: Non-synonymous FGD3 Variant as Positional Candidate for Disproportional Tall Stature Accounting for a Carcass Weight QTL (CW-3) and Skeletal Dysplasia in Japanese Black Cattle
Source: PLoS Genet. 2015 Aug 25;11(8):e1005433. doi: 10.1371/journal.pgen.1005433 (PMC4549114; doi:10.1371/journal.pgen.1005433)

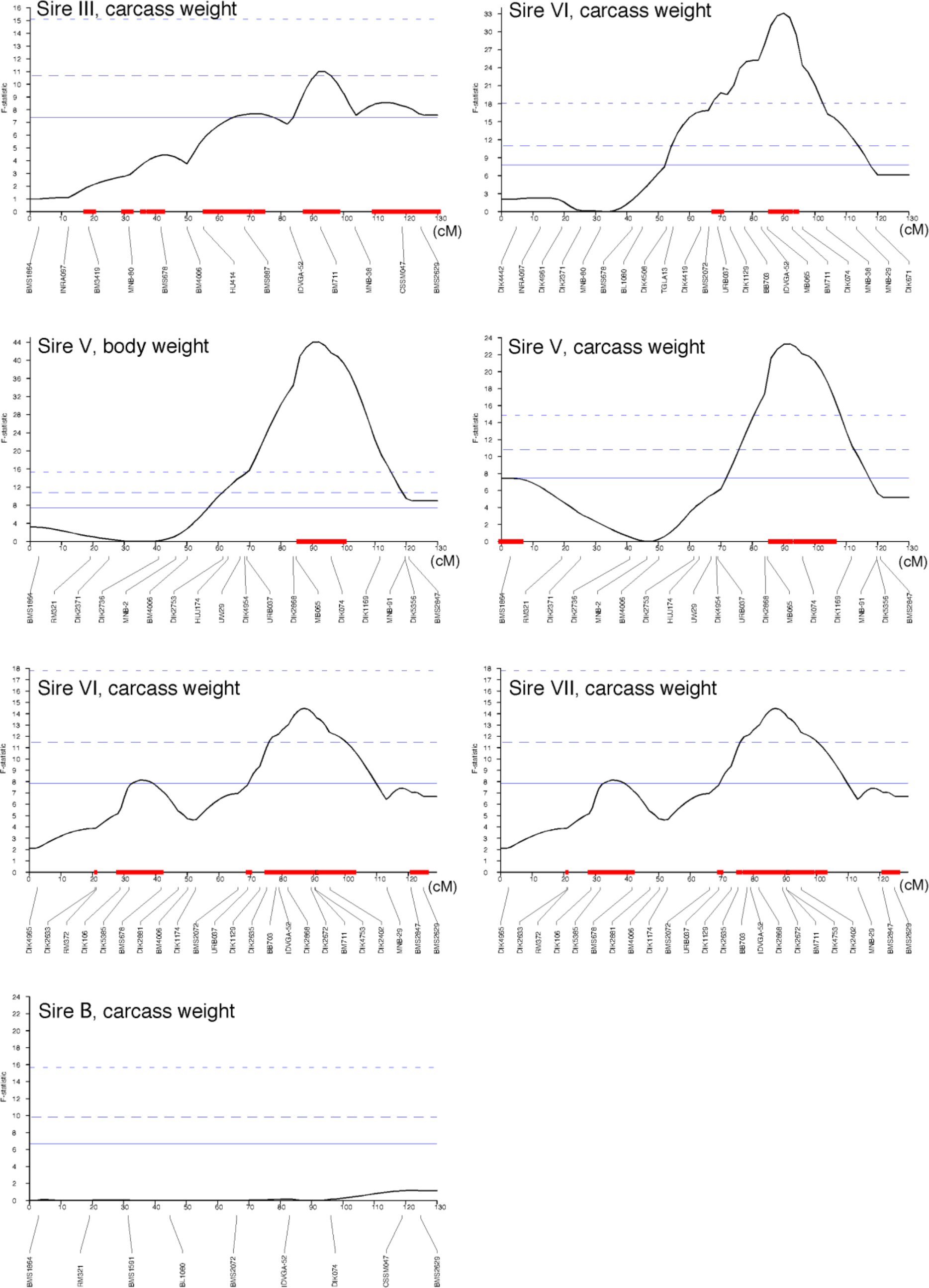

Supplement: S1 Fig — Summary of the quantitative trait locus (QTL) analyses is shown in S1 Table. Profiles for Sires I and II were described previously [7]. Marker locations were obtained from the Shirakawa-USDA linkage map [36]. Boxes on the x-axis indicate the 95% confidence interval of the QTL. Horizontal lines indicate the thresholds for chromosome-wise 0.1% (- --), 1% (––), and 5% (—–) significance levels. (TIF) [file pgen.1005433.s001.tif]

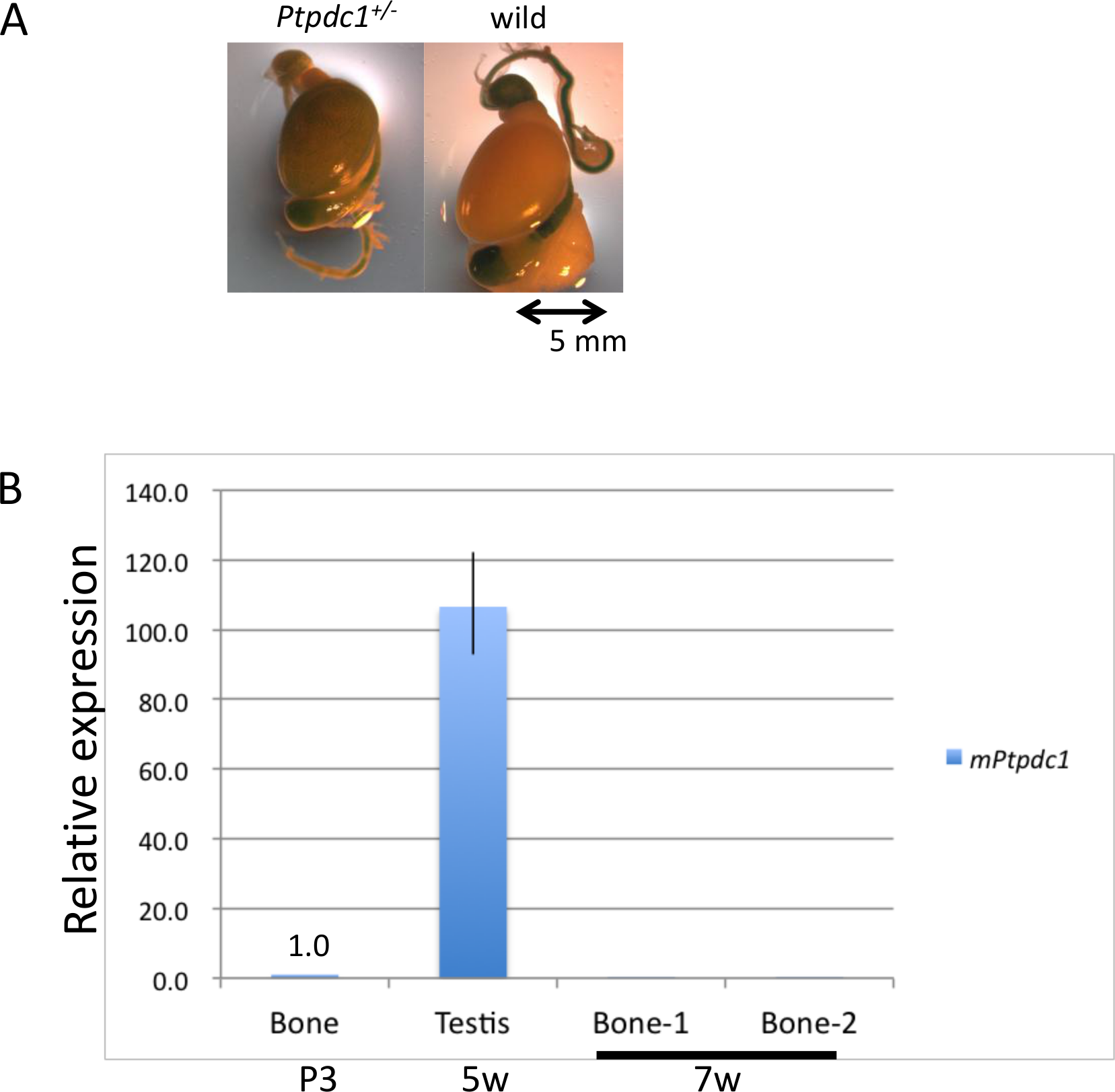

Supplement: S2 Fig — (A) X-gal staining of testes from Ptpdc1 +/- and wild-type littermates at 5-weeks of age. (B) Quantification of gene expression by real-time PCR. RNA was extracted from tibias and a testis of C57BL/6 mice. Real-time PCR was performed using TaqMan Gene Expression Assays (Applied Biosystems): Ptpdc1, Mm01327051_m1; Hprt, Mm01324427_m1. Hprt was used as an endogenous control. Data represent mean ± standard deviation (S.D.) in triplicate. Bone-1 and Bone-2 were from different individuals. (TIF) [file pgen.1005433.s002.tif]

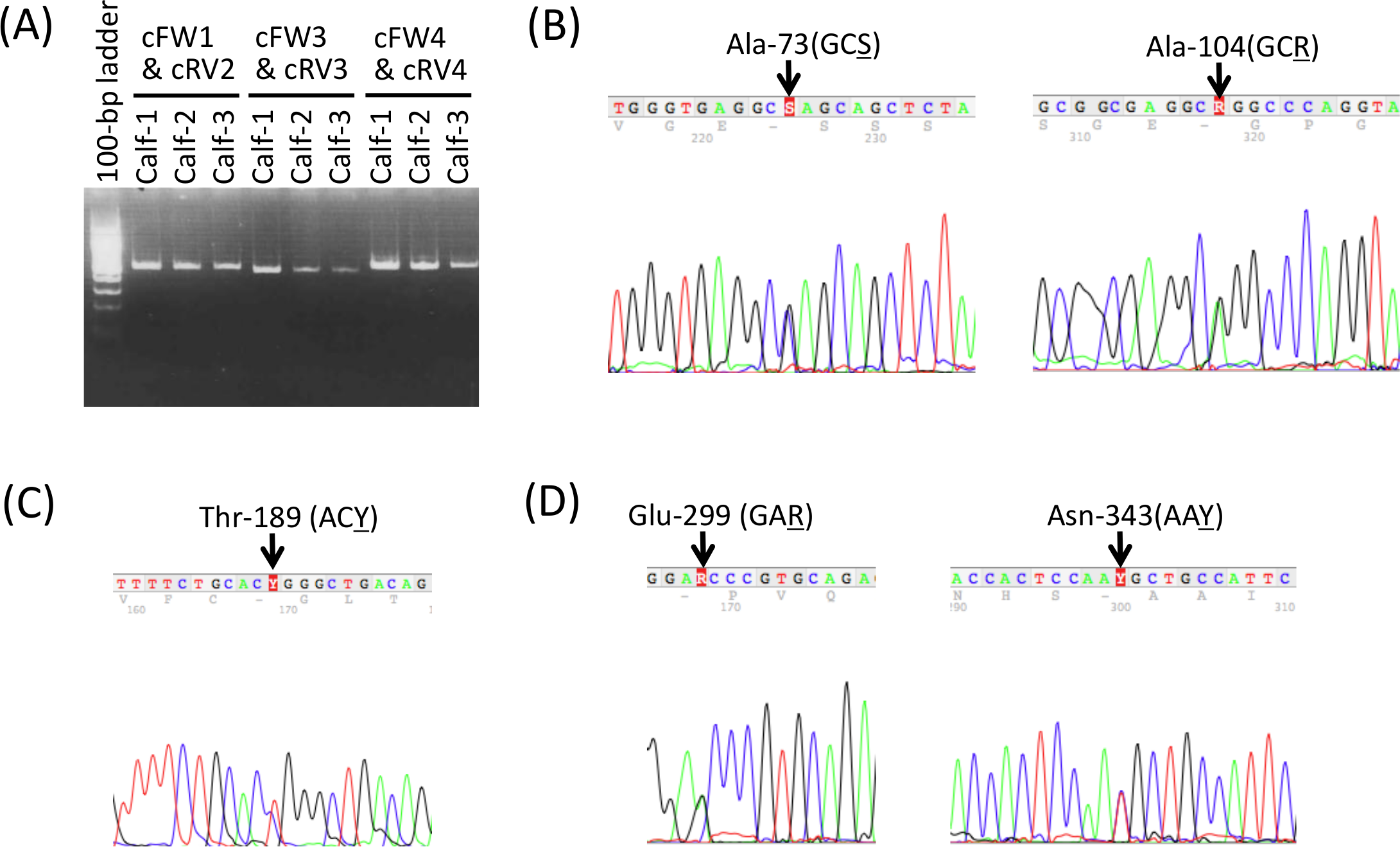

Supplement: S3 Fig — RNA was extracted from testes of three heterozygous calves using RNeasy (QIAGEN). RT-PCR was performed to amplify adjacent exons of a synonymous SNP. PCR primers are shown in S8 Table. PCR products were resolved in a 2% agarose gel and confirmed not to contain an additional band (A). A representative of the sequencing profile of the PCR products is shown from (B) to (D). In the panel (B), the read was converted to show the sense strand. (TIF) [file pgen.1005433.s003.tif]

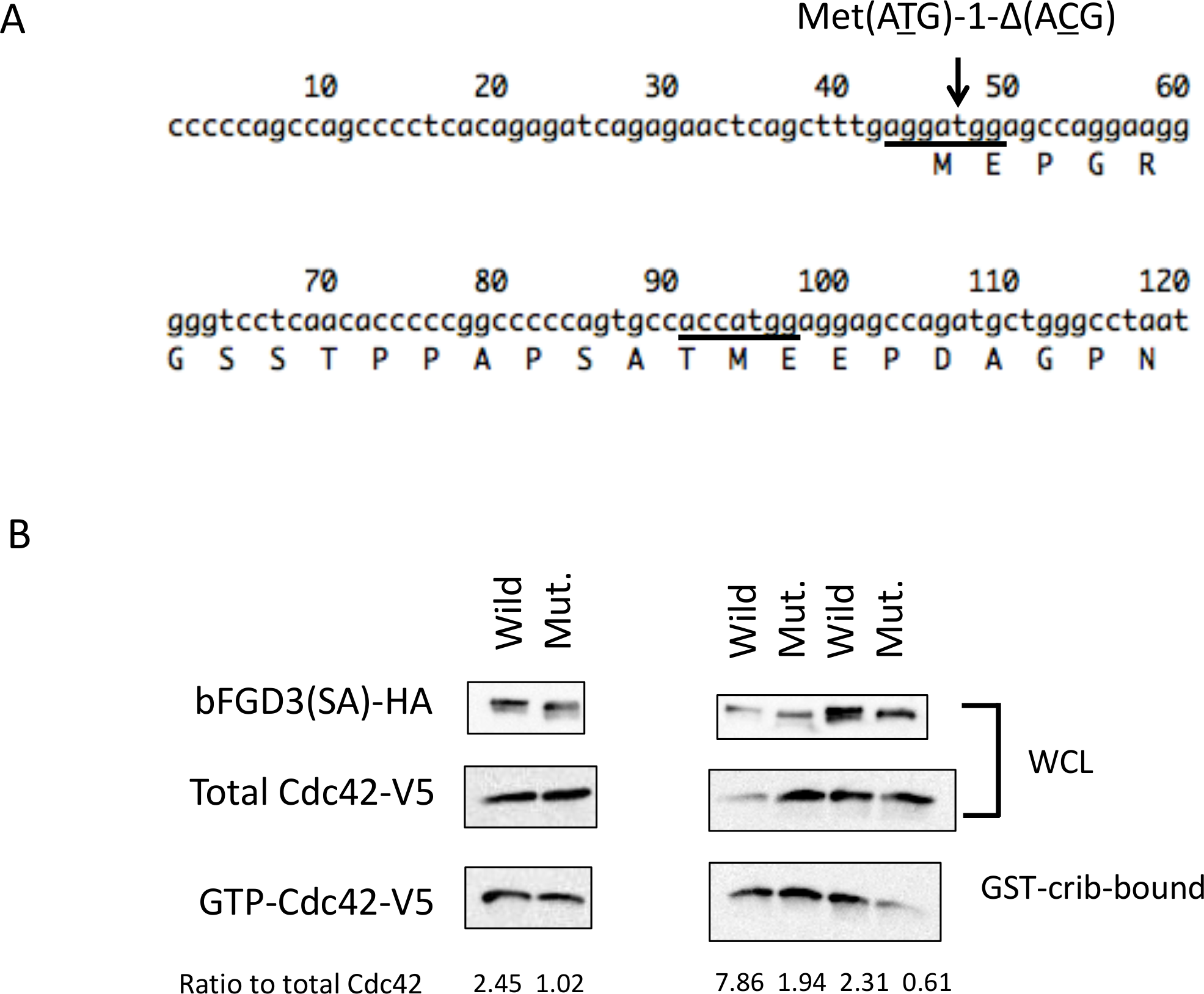

Supplement: S4 Fig — (A) The Kozak consensus sequence is present at the first and second methionine residues of bovine FGD3, shown by underlines. (B) The other two experiments of the GST–CRIB pull-down assay. (TIF) [file pgen.1005433.s004.tif]
